# mAPC-GibbsOS: an integrated approach for robust identification of gene regulatory networks

**DOI:** 10.1186/1752-0509-7-S5-S4

**Published:** 2013-12-09

**Authors:** Xu Shi, Jinghua Gu, Xi Chen, Ayesha Shajahan, Leena Hilakivi-Clarke, Robert Clarke, Jianhua Xuan

**Affiliations:** 1 Department of Electrical and Computer Engineering, Virginia Polytechnic Institute and State University, Arlington, VA, USA; 2 Departments of Oncology, Lombardi Comprehensive Cancer Center, Georgetown University, Washington, DC, USA

## Abstract

**Background:**

Identification of cooperative gene regulatory network is an important topic for biological study especially in cancer research. Traditional approaches suffer from large noise in gene expression data and false positive connections in motif binding data; they also fail to identify the modularized structure of gene regulatory network. Methods that are capable of revealing underlying modularized structure and robust to noise and false positives are needed to be developed.

**Results:**

We proposed and developed an integrated approach to identify gene regulatory networks, which consists of a novel clustering method (namely motif-guided affinity propagation clustering (mAPC)) and a sampling based method (called Gibbs sampler based on outlier sum statistic (GibbsOS)). mAPC is used in the first step to obtain co-regulated gene modules by clustering genes with a similarity measurement taking into account both gene expression data and binding motif information. This clustering method can reduce the noise effect from microarray data to obtain modularized gene clusters. However, due to many false positives in motif binding data, some genes not regulated by certain transcription factors (TFs) will be falsely clustered with true target genes. To overcome this problem, GibbsOS is applied in the second step to refine each cluster for the identification of true target genes. In order to evaluate the performance of the proposed method, we generated simulation data under different signal-to-noise ratios and false positive ratios to test the method. The experimental results show an improved accuracy in terms of clustering and transcription factor identification. Moreover, an improved performance is demonstrated in target gene identification as compared with GibbsOS. Finally, we applied the proposed method to two breast cancer patient datasets to identify cooperative transcriptional regulatory networks associated with recurrence of breast cancer, as supported by their functional annotations.

**Conclusions:**

We have developed a two-step approach for gene regulatory network identification, featuring an integrated method to identify modularized regulatory structures and refine their target genes subsequently. Simulation studies have shown the robustness of the method against noise in gene expression data and false positives in motif binding data. The proposed method has been applied to two breast cancer gene expression datasets to infer the hidden regulation mechanisms. The experimental results demonstrate the efficacy of the method in identifying key regulatory networks related to the progression and recurrence of breast cancer.

## Background

Living cells must be able to correctly respond to internal and external stimuli by adjusting gene expression levels [1]. Transcription factors (TFs) cooperatively regulate genes in forming gene regulatory networks, which plays a crucial role in the gene regulation process. Recently, biological researchers have shown that some diseases like cancer are closely related to the breakdown of regulatory networks, and many oncogenes (i.e., genes closely related to cancer) have been shown enrichment in this regulation mechanism [2]. Thus identification of transcriptional gene regulatory networks becomes a promising direction in the field of biology and bioinformatics. Several statistical methods such as principle component analysis (PCA) [3] and independent component analysis (ICA) [4] are developed to discover the underlying regulation mechanism. However, the strong assumption of independent or uncorrelated components cannot be easily satisfied in many real biological applications. Due to the fact that genes tend to cooperate to take effect, identifying co-expressed genes modules is an intuitive way to reconstruct regulatory networks. Therefore some clustering based methods such as Fuzzy C-means clustering [5] have been developed to discover co-expressed genes modules. However co-expressed gene modules are different from co-regulated genes in which we are interested. Co-regulated genes are regulated by some common TFs and tend to have similar gene expression pattern. On the contrary, co-expressed genes are not necessarily regulated by common TFs [6]. Moreover, these methods fail to incorporate the motif binding information provided by matching DNA upstream sequences and TFs with whole genome sequencing techniques [1].

Dynamic Bayesian Network [7] is one of the integrative methods, and it takes the motif-binding information as prior knowledge and learns the network from gene expression data. But the method will be hard to analyze data with large candidate TF pool, which limits its application to real biological studies. Network component analysis (NCA) [8] and several NCA-based methods such as FastNCA [9] are among several successful integrative methods, which are specifically developed to interpret gene regulatory network as a bipartite network. With some reasonable assumptions referred to as NCA criteria [8], NCA can decompose gene expression data to estimate the TF activity and then further infer the regulation strength. Nevertheless, motif binding data are often contaminated with many false positive connections and NCA is very sensitive to those false connections. To address the problem of false positive connections, Gu *et al*. have developed a regression based Gibbs sampling method (namely GibbsOS [10]) to discover true target genes from an initial gene pool. GibbsOS employs the same model as NCA does and summarizes regression t-test statistics into an outlier sum statistic [11], then with the help of Gibbs sampling strategy [12], it can identify true target genes from the gene pool. However, it fails to take modularized regulatory structure into consideration; therefore GibbsOS will perform poorly when a large number of TF candidates are investigated, which significantly limits its application to real biological studies.

The limitations of current methods can be summarized as follows: (i) being sensitive to contaminations (e.g., noise and false positives) in genomic data, (ii) failing to identify the modularized structure and (iii) being unable to handle a large number of candidate TFs. In this paper, we aim at tackling the above-mentioned limitations by proposing a novel method that combines a clustering method with GibbsOS to discover the hidden regulation mechanism; the clustering method is called motif-guided affinity propagation clustering (mAPC) [2], a modified version of affinity propagation clustering (APC) [13]. To evaluate the performance, we generate some synthetic data under different signal-to-noise ratios (SNRs) and numbers of false positive connections, with which to show that our method has an improved performance in regulatory network identification. Besides, two breast cancer patient datasets are used to demonstrate the feasibility of the proposed method for real biological studies. Experimental results show that the proposed method is able to identify active TFs and their target genes, hence, to reconstruct the underlying regulatory network.

## Results and discussion

### Motif-guided affinity propagation clustering and Gibbs sampler based on outlier sum statistics

**Figure 1:**
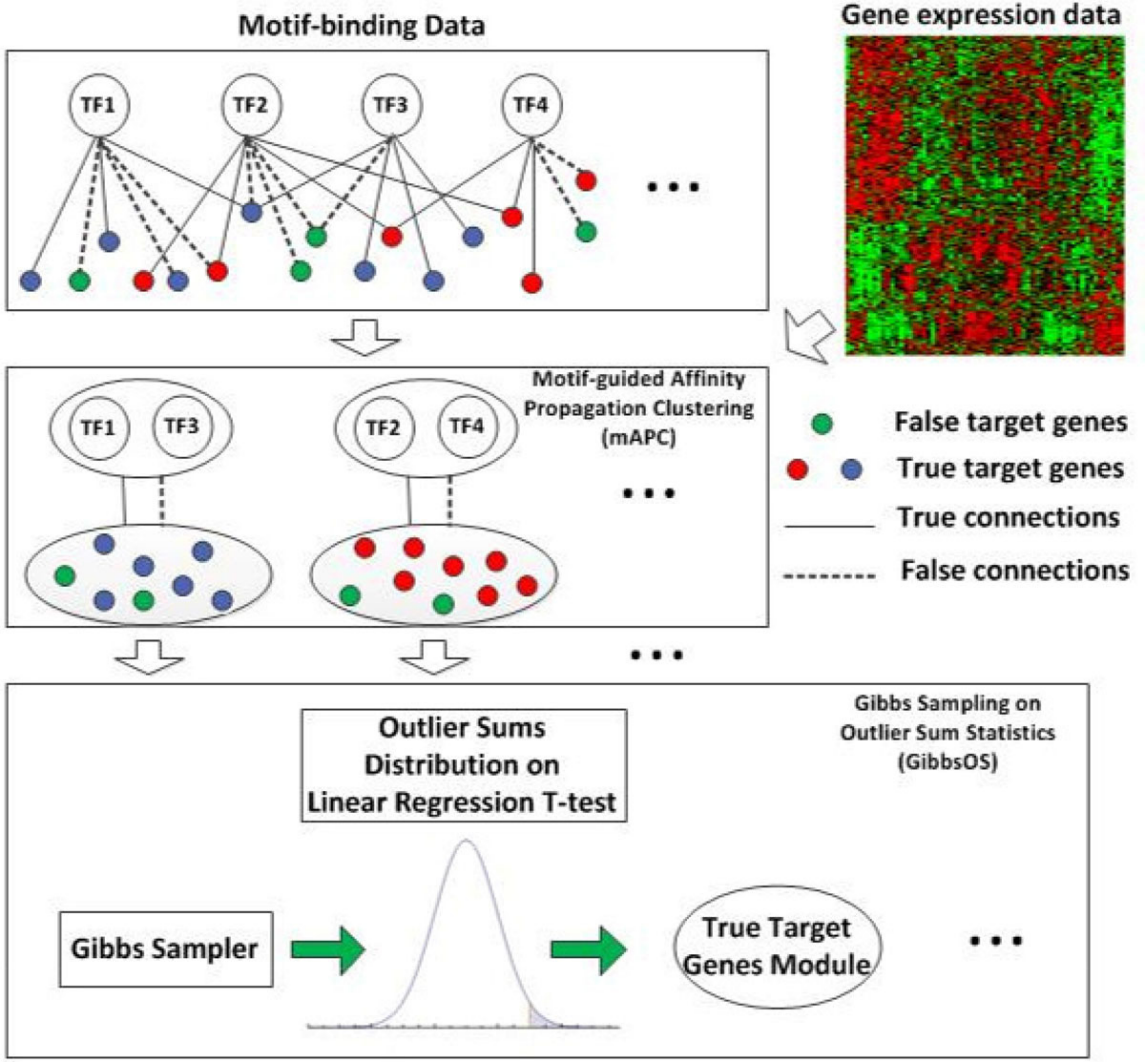
**Flow-chart of the proposed mAPC-GibbsOS approach**.

The flowchart of the proposed two-step method is shown in Figure 1. In the first step, mAPC is applied to identify the modularized structure by clustering genes into co-regulated modules. Unlike traditional clustering methods, mAPC uses both gene expression data and motif-binding data to measure the similarity between genes, which can reduce the noise effect from microarray gene expression data for more reliable clustering results. Besides gene clustering, TF identification is also applied within each cluster in the first step, in order to select the closely related TFs for further investigation.

In the second step, we apply GibbsOS to each cluster to remove false positive connections for target gene identification. For the convenience of explanation, we define true target genes as "foreground" genes and genes not regulated by TFs as "background" genes; in such a way, GibbsOS can be seen as identifying foreground genes from the entire gene pool. The detailed description of the method is summarized in the Methods section with mathematical details outlined.

### Simulation experiments

The simulation data are synthesized by MATLAB functions with 300 genes (which include 100 foreground genes and 200 background genes), 80 TFs and 20 experiments (or samples). The motif binding data are generated with modularized structures for both foreground genes and background genes, and the TF activities are randomly generated with Gaussian random variables of mean 0 and variance 1. Then the foreground gene expression data can be synthesized by a linear combination of motif-binding data and TF activities using a log-linear model provided by Liao *et al*. [8]. For the background genes, the gene expression data are randomly generated with Gaussian random variables (of mean 0 and variance 1) and the amplitude is modified to ensure the equal variance between foreground and background gene expression patterns. To perturb the data, noise is randomly added to gene expression data with certain signal-to-noise ratio (SNR). The level of false positives (FPs) added in motif binding data is measured by FP ratio, which is defined as the number of false positive connections over the number of true positive connections within foreground genes. To test the performance of the proposed method against noise in gene expression and false positives in motif binding data, we first fix the SNR level at 5 dB, and then test the performances of mAPC clustering and TF identification under three different FP ratios (0.5, 1.0 and 1.5). Further, we fix the FP ratio at 1.0 and generate simulation data under three SNR levels (0 dB, 5 dB and 10 dB) to assess the effect of false positives on the performance of mAPC-GibbsOS.

The performance evaluation is done systematically in terms of modularized structure reconstruction and target gene identification. Firstly, we use the simulation data to assess the performance of our clustering method, i.e., mAPC. A partition evaluation method, namely adjusted rand index (ARI) [14], is used here to compare the clustering results with the ground truth of the simulation data to assess the clustering accuracy (see Methods for details). With any two clusters, ARI can be calculated and summarized into a value between -1 and 1 and a higher value of ARI represents more similarity between the clusters. If the ARI value is 1, it means that the two clusters in comparison are exactly the same. Besides mAPC, three classical methods (which are k-means clustering, hierarchical clustering and APC) are used to compare and show the disadvantage of lacking motif binding information. Table 1 shows the ARI calculated under different SNRs and FP ratios. It can be seen that when SNR decreases from 10 dB to 0 dB, the performances of three competing methods tend to drop greatly. In contrast, with the support from motif binding information, mAPC can still maintain a good performance in clustering under low SNR cases. We can also see that mAPC cannot gain much improvement under high FP ratios, because when FP ratio is very large, motif binding information cannot provide strong support; thus for cases with high FP ratios, the trade-off parameter  λ (see Equation (4) in Methods) needs to be tuned to emphasize gene expression data more than motif binding information.

**Table 1:** Adjusted rand index values for clustering evaluation.

SNR(dB)	FP ratio	mAPC	APC	Hierarchical clustering	k-means clustering
10	1	**0.6363**	0.4039	0.3538	0.3504

	0.5	**0.7026**			
5	1	**0.5135**	0.2238	0.0495	0.1380
	1.5	**0.3067**			

0	1	**0.4037**	0.1088	0.0098	0.0921

Besides the clustering performance, the performance of TF identification (see Methods for details) is also needed to be evaluated; we use the area under the receiver operating characteristic (ROC) curve (AUC) as a criterion for evaluation. Table 2 shows the AUC values calculated under different simulation conditions. Considering Table 1 and Table 2 together, we can understand that the performance of TF identification is closely related to the accuracy of clustering, which implicitly underlines the importance of taking modularized structure into consideration. Under different experimental conditions, the performance of TF identification is excellent with AUC values above 0.83; it supports that the modularized structure can be robustly reconstructed by mAPC-GibbsOS.

**Table 2:** AUC values for mAPC-based TF identification.

SNR(dB)	FP ratio	mAPC	
		Cluster1	Cluster2
10	1	0.9844	0.9838

	0.5	0.9650	0.9656
5	1	0.9225	0.9225
	1.5	0.8369	0.8313

0	1	0.8850	0.8850

*Cluster 1 and 2 are not the same cluster under different conditions

After evaluating the performance of clustering and TF identification (in revealing modularized structures), we need to further test the performance of target gene identification. Similar to mAPC performance evaluation, both gene expression noise and false positive connections are taken into consideration to investigate their effects on the performance. The experimental results are also summarized by AUC and the values are shown in Table 3. Under the same FP ratio at 1.0, our proposed method has an AUC improvement of 0.15 in average for each SNR level. From another point of view, when SNR is fixed at 5 dB, mAPC-GibbsOS can achieve an average improvement of 0.1 under different FP ratios.

**Table 3:** AUC values of target gene identification of mAPC-GibbsOS vs. GibbsOS.

SNR(dB)	FP ratio	mAPC-GibbsOS		GibbsOS
		Cluster1	Cluster2	
10	1	**0.8437**	**0.8486**	0.6568

	0.5	**0.8296**	**0.8021**	0.7099
5	1	**0.7625**	**0.8417**	0.6290
	1.5	**0.7679**	**0.7397**	0.5952

0	1	**0.7203**	**0.8243**	0.6102

*Cluster 1 and 2 are not the same cluster under different conditions

**Figure 2:**
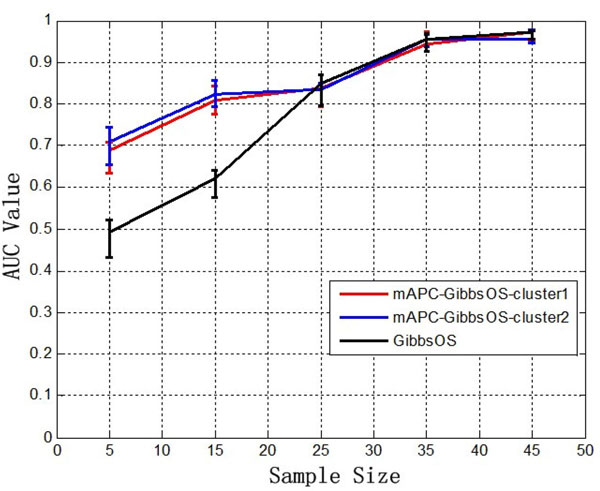
**AUC values of performance evaluation under different sample sizes**.

It is also worth noting that the small sample size of gene expression data is a common problem for robust identification of gene regulatory networks. In order to study the impact of the small sample size onto the performance of our proposed method, we have generated gene expression data and motif-binding data under 5 different sample sizes (with 5, 15, 25, 35 and 45 samples) with 300 genes and 80 TFs. The noise is generated under standard normal distribution with a fixed SNR at 5 dB. FP ratio for motif-binding data is set to 0.5. Then AUC values of both mAPC-GibbsOS and GibbsOS are calculated 5 times for each condition. The experimental results are shown in Figure 2 and Table 4. It can be seen from Figure 2 that the small sample size does impact the performance of the methods; in particular, when the number of samples is less than 25, the performance degrades for both mAPC-GibbsOS and GibbsOS. However, the performance degradation of mAPC-GibbsOS is much less than that of GibbsOS; as a matter of fact, mAPC-GibbsOS outperforms GibbsOS with an increase AUC of 0.18 to 0.2, when the sample size is smaller than 25. To our understanding, that mAPC-GibbsOS suffers less from the small sample size problem than GibbsOS may largely be a result of its consideration of the underlying modularized structure. By considering the underlying modularized structure, the proposed method is capable of dividing the data into two or more modules with reduced data dimension by gene clustering and TF identification. In our case, the data is divided into two modularized parts for the method to identify target genes; as a result, the target gene identification using mAPC-GibbsOS can achieve an AUC value over 0.7, which is 0.2 higher than that of GibbsOS.

**Table 4:** AUC values of target gene identification under different sample sizes.

			Sample Size				
			5	15	25	35	45
		Max	0.7077	0.8421	0.8704	0.9724	0.9742
	**Cluster1**	Median	0.6886	0.8090	0.8381	0.9435	0.9732
		Min	0.6333	0.7765	0.7937	0.9268	0.9492
**mAPC-GibbsOS**							
		Max	0.7456	0.8563	0.8497	0.9678	0.9769
	**Cluster2**	Median	0.7082	0.8231	0.8338	0.9554	0.9534
		Min	0.6549	0.7953	0.7957	0.9383	0.9460

		Max	0.5223	0.6405	0.8704	0.9687	0.9742
	**GibbsOS**	Median	0.4933	0.6200	0.8497	0.9554	0.9732
		Min	0.4313	0.5759	0.7957	0.9268	0.9534

*Cluster 1 and 2 are not the same cluster under different conditions

### Breast cancer microarray data

Our method is further tested upon two estrogen receptor (ER) related breast cancer patient datasets mentioned in Symmans *et al*. [15] and Loi *et al*. [16] to identify gene regulatory networks. The patient samples in the two datasets are divided into 'early recurrence' group (< 3 years) and 'late recurrence' group (> 6 years) according to survival time. The Symmans *et al*. dataset [15] consists of 21 samples in 'early recurrence' group and 41 samples in 'late recurrence' group, and the Loi *et al*. dataset [16] has 49 samples in 'early recurrence' group and 76 samples in 'late recurrence' group. An initial gene set is selected by T-test on gene expression data between 'early recurrence' and 'late recurrence' groups with a threshold p-value of 0.05. In this study, we analyze the up-regulated genes (over-expressed in 'early recurrence' group) and down-regulated genes (over-expressed in 'late recurrence' group) separately. For Symmans *et al*. data [15], totally 615 up-regulated genes and 344 down-regulated genes are selected, while there are 668 up-regulated genes and 559 down-regulated genes selected for Loi *et al*. data [16]. Motifs are selected from ER related signaling pathways and binding sites [17], which are believed to have strong connections with cancer progression. Finally 88 and 84 motifs are chosen for Symmans *et al*. data [15] and Loi *et al*. data [16] respectively.

**Figure 3:**
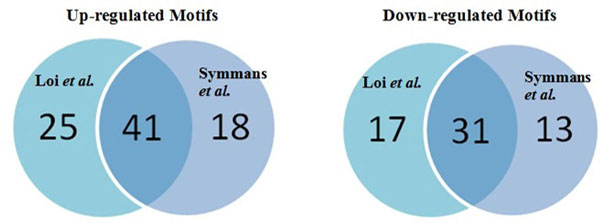
**Venn diagrams of identified motifs from Symmans *et al*. data **[15]** and Loi *et al*. data **[16].

**Figure 4:**
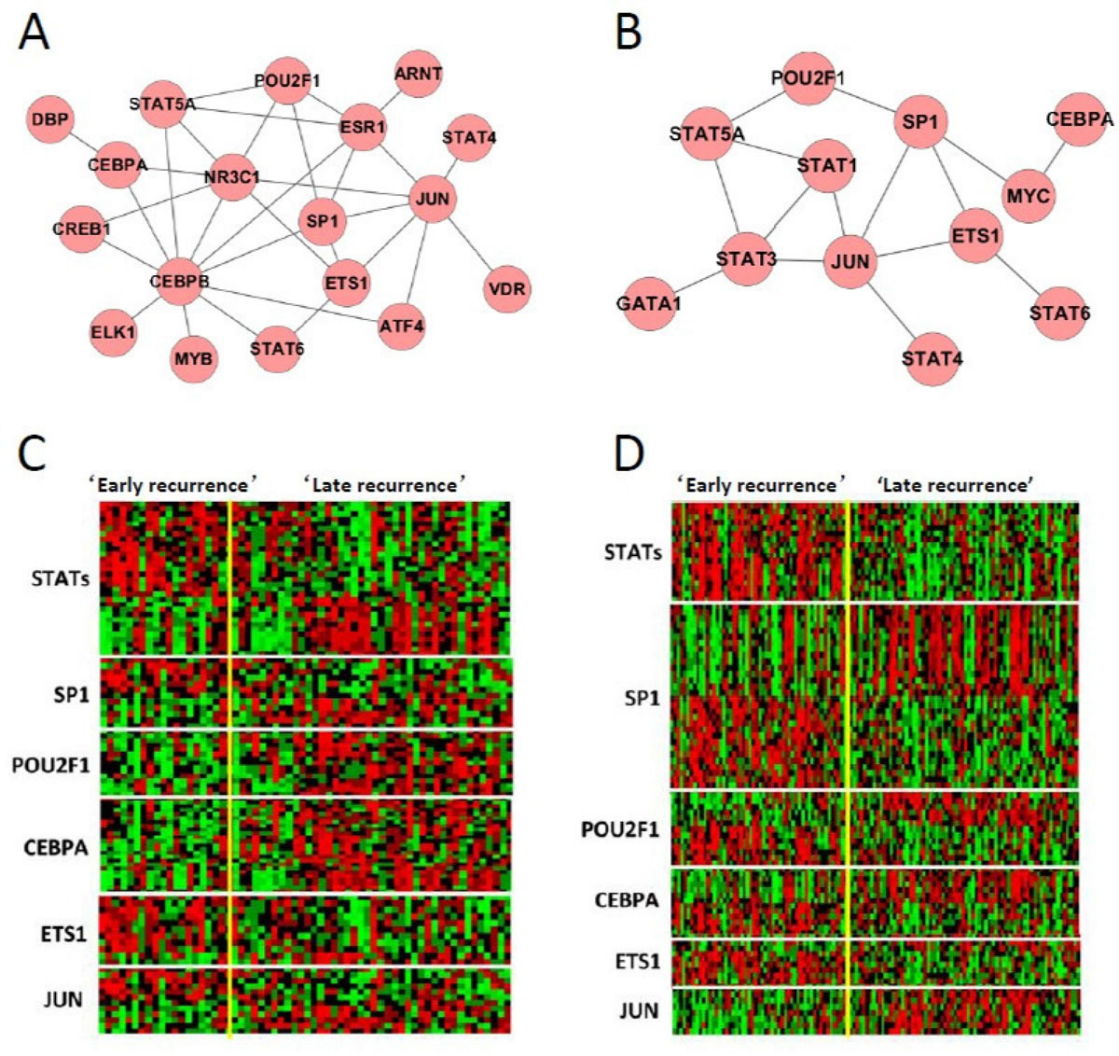
**Identified TF networks (as connected by protein-protein interactions)**: (A) up-regulated TFs, (B) down-regulated TFs, **and expression patterns of target genes of selected TFs on**: (C) Symmans *et al*. data [15] and (D) Loi *et al*. data [16].

Applying the proposed mAPC-GibbsOS method to the two breast cancer datasets, we can find consistent results in the TF layer. The analysis is done for up-regulated genes and down-regulated genes separately; for each case, we identify two clusters of genes and then motif enrichment is conducted within each cluster. In Figure 3, the Venn diagrams show that totally 66 up-regulated motifs and 48 down-regulated motifs are identified from Loi *et al*. data [16], and 59 up-regulated motifs and 44 down-regulated motifs are identified from Symmans *et al*. data [15]. About 65% of the motifs are shared among these two datasets; this large overlap indicates that the similarity between the regulation mechanisms enriched in these two datasets is high. Furthermore, in order to further study the overlap motifs of the two datasets, we have matched the enriched motifs to corresponding TFs and incorporated the protein-protein interaction information from HPRD database [18]. Then we can construct TF networks with protein-protein interactions under each case to help study the regulation mechanism. Figure 4(a) and 4(b) show the TF networks connected by protein-protein interaction in terms of up- and down-regulated networks, respectively. It can be seen that there is a large overlap between the two networks and the common genes such as JUN, STAT5A and SP1 have already been shown the involvements in cancer progression. JUN and CREB play an important role in MAPK signaling pathway and MAPK pathway has been shown as the center of some signaling networks controlling the growth, proliferation and differentiation of many cell types [19]. Furthermore, JUN is closely related to drug resistance [20] and CREB is a cyclic-AMP response element-binding protein and its over-expression or activation is frequently observed in breast cancer tissues [21]. Besides, CEBPA/B TFs have also been shown a strong correlation with cancer cell growth and differentiation [22]. STAT family TFs such as STAT5 play a central role in Jak-STAT signaling pathway whose importance has already been stressed in [23]. The involvement of POU2F1 in hormonal signals [24] can also lead to cancer progression, and SP1 is demonstrated to be one of the TFs that either enhance or repress the activity of promoters of genes involved in differentiation, cell cycle progression and oncogenesis [25]. ETS1 and ELK1 are members of ETS family genes that are often over-expressed in breast cancer [26]. In Figure 4(c) and 4(d), the gene expression patterns for identified target genes of the overlapped TFs in (a) and (b) are shown on Symmans *et al*. data [15] and Loi *et al*. data [16] respectively. We can see that the target genes show both up-regulated and down-regulated patterns, because corresponding TFs involve in both regulation directions. If considering the up-regulation and down-regulation separately, we can observe consistent expression patterns, which further support the involvement of identified TFs in regulation.

**Figure 5:**
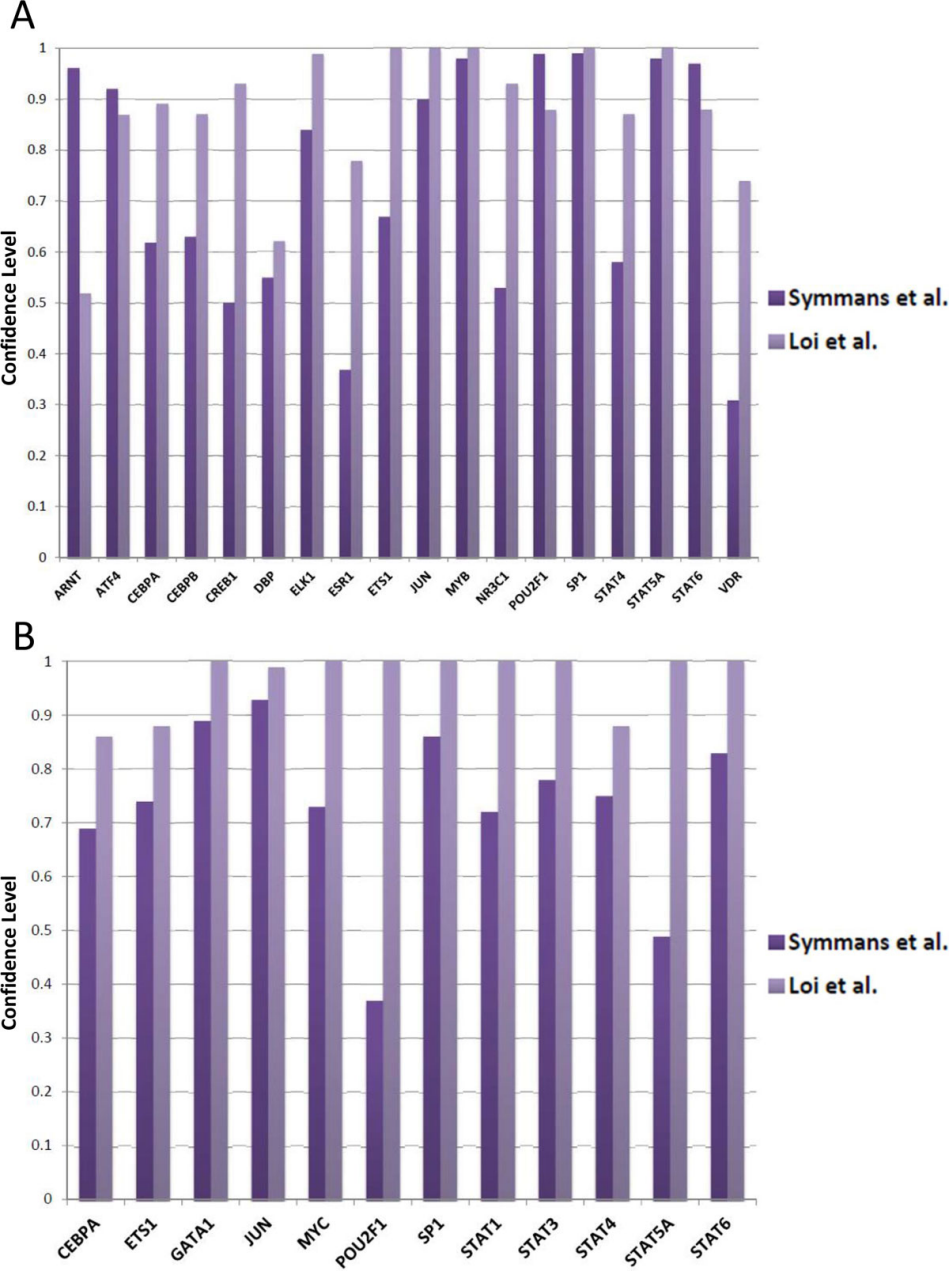
**Bootstrapping confidence scores of identified TFs**: (A) up-regulated TFs, and (B) down-regulated TFs.

**Figure 6:**
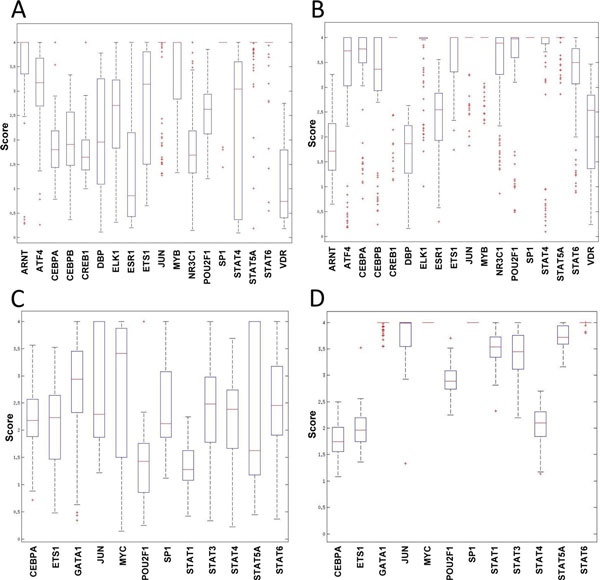
**Bootstrapping score variation for up-regulated TFs**: (A) Symmans *et al*. data [15], (B) Loi *et al*. data [16]**and down-regulated TFs**: (C) Symmans *et al*. data [15], (D) Loi *et al*. data [16].

To further demonstrate the robust performance of mAPC-GibbsOS, a bootstrapping procedure is used in this breast cancer study to provide the confidence level of the identified TFs. The rationale behind this approach is that the TFs with greater frequency included in the identified networks should be more confident. The non-parametric bootstrapping process generates different datasets many times by re-sampling the experimental samples with replacement. Our proposed method, mAPC-GibbsOS, is applied on each newly generated data, and the times are counted for TFs identified with a certain confidence level (which is set to 0.9 in this study). Totally 100 bootstrap versions of the gene expression data are used in this bootstrapping analysis; the results are summarized in terms of confidence score, which is calculated as the frequency of TFs (shown in Figure 5). As seen from Figure 5, all the identified TFs have a confidence score greater than 0.3 and most of them appear more than 50 times. It can also be seen that some TFs such as CEBPA, ETS1, JUN, SP1 and STAT family TFs are identified confidently by mAPC-GibbsOS in both up-regulated and down-regulated networks. Furthermore, these two regulation networks also have some specific TFs like ATF4, CEBPB, ELK1, MYB and POU2F1 in up-regulated network and MYC in down-regulated network. We have further converted the p-value (as calculated by Equation (5) in the Methods section) from TF identification to a score, which is the inverse cumulative distribution function (ICDF) of the corresponding p-value under standard normal distribution. The upper limit of the score is set to 4 to remove some extreme values calculated by the ICDF. Figure 6 shows the variation of the score in terms of boxplot. Most TFs shown have a median value larger than 1.65 which is the score threshold under confidence level 0.9. Due to the cut-off threshold at 4, some strong TF only shows a bar in the box plot, which means that over 75% of the scores reach the upper limit. As seen from Figure 6, TFs identified on Loi *et al*. data [16] have very high and stable score distributions. For Symmans *et al*. data [15], the variation is a little larger, but most of the scores vary in the high confident region.

## Conclusion

In this paper, we have proposed a new method consisting of a clustering method (i.e., mAPC) and a sampling based method (i.e., GibbsOS) to tackle the problem of regulatory network identification. mAPC is different from traditional clustering methods in terms of constructing co-regulated gene modules by utilizing both microarray gene expression data and motif binding information. Following mAPC, GibbsOS is applied to refine the module for target gene identification to solve the issue of false positive connections in motif binding data.

The proposed method is tested by simulation data with different SNRs and FP ratios. Significant improvements have been observed in terms of both gene module identification and target gene identification. To further test the method with real biomedical applications, two breast cancer patient datasets are used for the identification of regulatory networks related to recurrence of breast cancer. As a result, a key set of regulatory networks has been reconstructed with active transcription factors and their target genes. Importantly, these regulatory networks are functionally enriched in the progression and recurrence of breast cancer, warranting further investigations to assess their functional roles by biological experiments.

## Methods

mAPC-GibbsOS is an integrative method that focuses on identifying modularized gene regulatory network. We use the log-linear model proposed by Liao *et al*, [8]

(1) X=AS+Γ,

where  X is an N×K matrix representing the measured gene expression data,  A is the regulation strength matrix with a dimension of N×M, M×K matrix  S specifies the TF activities,  Γ represents the inevitable experimental noise, N is the number of genes, K is the number of experiments and M is the number of TFs. This model interprets the regulatory mechanism as a bipartite network and the expression of gene can be considered as a direct result from TF activity associated with related regulation strength. Based on this model, we can divide the whole gene set into two distinct categories: (1) "foreground" genes that are truly regulated by TFs and (2) "background" genes that are not related with TFs. It can also be seen that only the foreground genes will hold the relationship between gene expression and TF activity, therefore, it is necessary to identify modularized structure on the foreground gene set rather than the whole gene set.

### Motif-guided affinity propagation clustering (mAPC)

Our first goal is to use clustering methods to group the genes regulated by common TFs. Recently one innovative clustering method called affinity propagation clustering (APC) [13] has been developed, which has shown promising results for clustering data points. APC iteratively passes messages between each pair of nodes and generates several 'exemplars' that show the characteristics of their own clusters best. The messages passed include "responsibility" and "availability" and they together indicate how appropriate to choose a point as exemplar. The application of APC in clustering of gene expression data usually uses a Euclidean distance to measure similarity,

(2) $$\text{s}\left(\text{i},\text{j}\right)=-{\left|\left|x_{i}-x_{j}\right|\right|}^{2},$$

where s(i,j) is the similarity between gene  i and  j, $x_{i},x_{j}$ are the gene expression data of gene  i and  j. It implies that the larger Euclidean distance between gene  i and  j is, the less similarity between them is. However, this cost function only considers clustering genes with similar gene expression patterns, thus it can only cluster co-expressed genes rather than co-regulated genes. Since gene expression data are relatively noisy, this cost function tends to make the clustering results not reliable. In order to solve this problem, we propose a new cost function to incorporate motif binding information. Suppose we have a binding strength matrix  W with the same dimension of the regulation matrix  A (N×M) showing the possible connections between N genes and M TFs. w(i,j) will be a value taking either 0 or 1 representing the possible binding connection between gene  iand TF  j. Given a candidate TF j, the probability that gene k and t are simultaneously regulated by TF  j is proportional to w(k,j)×w(t,j). Hence, considering all available M TFs, the co-regulation probability between gene k and t from motif binding data will be proportional to

(3) $$s_{reg}\left(k,t\right)=\sum_{j=1}^{M}w\left(k,j\right)\times w\left(t,j\right).$$

Incorporating co-regulation information described in Equation (3) into the classical similarity measurement, we can formulate our new cost function for APC as:

(4) $$\text{s}\left(\text{i},\text{j}\right)=-\left(1-\lambda \right){\left|\left|x_{i}-x_{j}\right|\right|}^{2}+\lambda \;s_{reg}\left(i,j\right),$$

where  λ is a trade-off parameter between 0 and 1 to adjust the contribution of gene expression data and that of motif binding information. If  λ is 1, the clusters generated by mAPC will totally depend on motif binding information. On the contrary, if  λ is 0, mAPC turns out to be the classical APC as applied to gene expression data alone. As gene expression data are noisy, the second term can lower the noise effect by the positive support from binding information. On the other hand, the false connections existed in matrix  W will be penalized by large negative gene expression similarity measurement, because genes not co-regulated do not share similar gene expression patterns. In general, this type of balanced cost function will provide us a better representation of gene modules in terms of both co-regulation and co-expression.

### Transcription factor identification

After obtaining clusters of genes, we have already uncovered the modularized structure for genes, but we need to further associate clusters of genes with TFs, which can be accomplished by testing TF enrichment for every cluster. A hyper-geometric test is applied to each TF to tackle this problem with the following hypotheses:

$${\text{H}}_{0}:TF\;j\;is\;not\;enriched\;in\;cluster\;c;{\text{H}}_{1}:TF\;j\;is\;enriched\;in\;cluster\;c.$$

Assume that there are ${\text{N}}_{\text{c}}$ genes in cluster c and ${\text{N}}_{\text{b}}$genes have connections with TF j. In the whole gene population, the total number of genes is N and that of TF j related genes is ${\text{N}}_{\text{B}}$. Then we generate a null distribution by randomly sampling all the N genes to form random clusters with size ${\text{N}}_{\text{c}}$ many times. For each cluster, we count the number of genes regulated by TF j, denoted as n. We can then calculate p-value as the percentage of the clusters which have a number n greater than ${\text{N}}_{\text{b}}$. In fact, this process can be modelled by a hyper-geometric distribution and the p-value can be calculated as follows:

(5) $$\text{p}-\text{value}=\overset{\min\left(N_{B},N_{c}\right)}{\underset{i=N_{b}}{\sum }}\left(\begin{array}{c} N_{B}\\ i \end{array}\right)\left(\begin{array}{c} N-N_{B}\\ N_{c}-i \end{array}\right)/\left(\begin{array}{c} N\\ N_{c} \end{array}\right).$$

Based on the calculated p-value, we can determine the enrichment of TFs in different clusters with a pre-defined threshold (which can be adjusted according to various cases).

### Gibbs sampler based on outlier sum statistics (GibbsOS)

GibbsOS is a method that exploits both gene expression data and motif binding information to identify foreground genes from a possible large pool of genes. Firstly, suppose we can find a foreground gene set $\Theta =\left[\theta_{1},\theta_{2},\ldots ,\theta_{\text{M}}\right]$ as "seed" genes, where  M seed genes are selected for each of  M TFs. Then according to Equation (1),

(6) $$E_{\Theta }=A_{\Theta }S,$$

where $E_{\Theta }$ is the gene expression of  M seed genes, $A_{\Theta }$ refers to the binding matrix of the seed genes and  S is the corresponding TF activity. Solving the Equation (6) for  S,

(7) $$S={\left(A_{\Theta }\right)}^{-1}E_{\Theta }=\alpha E_{\Theta }.$$

Given another foreground gene  y,

(8) $$E_{y}\;=\;A_{y}S\;=\;A_{y}\alpha E_{\Theta }\;=\;\beta E_{\Theta },$$

where $E_{y}$ and $A_{y}$ are the gene expression and binding matrix of gene y and $\beta =A_{y}\alpha$ infers the relationship between gene y and the seed genes. If gene y is a foreground gene truly regulated by TF  j, then $\beta_{j}$ should be not equal to 0, due to the similarity shared between gene y and seed gene of TF  j. Therefore, the regulation relationship between gene  y and TF  j can be solved by linear regression hypothesis test on the parameter estimated,

$${\text{H}}_{0}:\beta_{\text{j}}=0;{\text{H}}_{1}:\beta_{\text{j}}\neq 0.$$

By iteratively conducting the test for gene y and all the TFs, the regulation mechanism of gene y can be determined. The estimation of  β, denoted as $\hat{\beta }$, can be obtained by least square estimation,

(9) $$\hat{\beta }={\left(F^{T}F\right)}^{-1}F^{T}y=CF^{T}y,$$

where we replace $E_{y}$ and $E_{\Theta }$ by  y and  F for the convenience of representation. The mean squared error (MSE) of this fitted model with  K experiments and  M TFs is

(10) $$\text{MSE}=\frac{{\left(y-F\hat{\beta }\right)}^{\text{T}}\left(y-F\hat{\beta }\right)}{degree\;of\;freedom}=\frac{y^{T}y-y^{T}FCF^{T}y}{\text{K}-\text{M}-1},$$

where degreeoffreedom is K-M-1. Then a significant test statistic of regression coefficients can be applied,

(11) $$\text{t}=\frac{{\hat{\beta }}_{\text{j}}}{\sqrt{\text{MSE}}\cdot \sqrt{{\text{C}}_{\text{jj}}}},$$

where ${\text{C}}_{\text{mm}}$ is the m-th diagonal element of the matrix  C. This test statistic t will follow a Student t-distribution with a degree of freedom of K-M-1 , and then we can obtain corresponding p-value to make decision on hypothesis with certain predefined confidence level.

Although we have already demonstrated the method to identify foreground genes, we actually do not know the ground truth behind the data. It is impossible to accurately draw foregrounds as seed genes, but we are sure that there must be multiple true foreground genes in the pool, thus we can make those foreground genes support each other. This approach can be completed in an iterative way, assuming that the candidate genes for TF j can be divided into $\Phi_{\text{jF}}$ and $\Phi_{\text{jB}}$ ($\Phi_{\text{jF}}\cap \Phi_{\text{jB}}=\emptyset$ and $\Phi_{\text{jF}}\cup \Phi_{\text{jB}}=\Phi_{\text{j}}$), two sets containing foreground genes and background genes respectively. To start the iteration, we randomly select one gene $\theta_{\text{ji}}$$\left(1\leq \text{i}\leq {\text{K}}_{\text{j}}\right)$ from $\Phi_{\text{j}}$, where ${\text{K}}_{\text{j}}$ is the cardinality of gene set $\Phi_{\text{j}}$. Together with the candidate genes for other TFs, we can obtain a foreground gene list $\Theta =\left[\theta_{1},\theta_{2},\ldots ,\theta_{\text{j}}=\theta_{\text{ji}},\ldots ,\theta_{\text{M}}\right]$. Then we apply the linear regression T-test mentioned in Equation (11); totally ${\text{K}}_{\text{j}}-1$ ($\theta_{\text{j}1}$ to $\theta_{\text{j}{\text{K}}_{\text{j}}}$ except $\theta_{\text{ji}}$) t statistics will be generated for TF j.

In order to obtain a summarized score for choosing $\theta_{\text{j}}=\theta_{\text{ji}}$, we use an outlier sums (OS) statistic and it can be defined as,

(12) $$\text{OS}=\overset{K_{j}}{\underset{k\neq i}{\sum }}\left|t_{\text{jk}}\right|\cdot \text{I}\left(\left|t_{\text{jk}}\right|-t_{\frac{\alpha }{2},K-M-1}\right),$$

where ${\text{t}}_{\text{jk}}$ is the t-test statistic of gene k under the condition that $\theta_{\text{j}}=\theta_{\text{ji}}$, and I(⋅) is an indicator function which outputs 1 with a non-negative value and outputs 0 otherwise, and $t_{\frac{\alpha }{2},K-M-1}$ refers to the threshold of  α confidence level with K-M-1 degree of freedom. As interpreted from the above equation, if $\theta_{\text{ji}}$ is a good choice of foreground genes for TF j, OS will achieve a large value. On the contrary, if $\theta_{\text{ji}}$ is not supported by other genes, OS will have a very small value or even 0. Considering Equation (12) as a function of $\theta_{j}$ given the choices of other TFs, we can then rewrite it as:

(13) $$\text{O}{\text{S}}_{\theta_{\text{j}}}=f\left(\theta_{j}|\theta_{1},\ldots ,\theta_{\text{j}-1},\theta_{\text{j}+1},\theta_{\text{M}}\right).$$

The form of OS statistic indicates the dependency of the decision of one TF on the choices of foreground genes for other TFs, but what we are interested in is the marginal function which is independent of the choices of other TFs.

Gibbs sampler is a fairly good technique which can draw samples with respect to marginal distribution with only the conditional distribution. For our GibbsOS case, we can construct a conditional probability density function as follows:

(14) $$p\left(\theta_{j}|\theta_{1},\ldots ,\theta_{\text{j}-1},\theta_{\text{j}+1},\theta_{\text{M}}\right)=\frac{1}{K_{0}}f\left(\theta_{\text{j}}|\theta_{1},\ldots ,\theta_{\text{j}-1},\theta_{\text{j}+1},\theta_{\text{M}}\right),$$

where the function f(⋅) is the same function as Equation (13) (i.e., the outlier sum statistic function) and ${\text{K}}_{0}$ is a normalization constant which ensures the total probability equals one. Then after initializing a set of candidate genes $\left[\theta_{1}^{0},\theta_{2}^{0},\ldots ,\theta_{\text{M}}^{0}\right]$, we can sample the genes as:

(15) $$\theta_{\text{j}}^{t+1}\sim p\left(\theta_{\text{j}}{|\theta }_{1}^{t+1},\ldots ,\theta_{\text{j}-1}^{t+1},\theta_{\text{j}+1}^{t},\theta_{\text{M}}^{t}\right),$$

where t denotes the *t*-th sampling iteration. At each iteration step, we sequentially sample one candidate foreground gene for each TF once. When going through sufficient steps, we not only sample those candidate genes, but also estimate the marginal distributions. In our case, we can simply use the frequency of a gene emerged in the sampled sequence to approximate the empirical marginal distribution. Then the genes with higher frequency will be more probable to be foreground genes.

### Adjusted rand index for performance evaluation

To evaluate the clustering performance, a partition evaluation method, namely adjusted rand index (ARI) [14], is used in this study. We will compare the results with the ground truth of the simulation data to assess the clustering accuracy. Suppose the resulting  N clusters are $\text{C}=\left\{{\text{c}}_{1},{\text{c}}_{2},\ldots ,{\text{c}}_{\text{N}}\right\}$ and the  M ground truth clusters are $\text{G}=\left\{{\text{g}}_{1},{\text{g}}_{2},\ldots ,{\text{g}}_{\text{M}}\right\}$. Let ${\text{n}}_{\text{ij}}$ be the number of elements existed in both cluster ${\text{c}}_{\text{i}}$ and ${\text{g}}_{\text{j}}$, and ${\text{n}}_{\text{i}}.$and ${\text{n}}_{\text{.j}}$ are the total numbers of genes in ${\text{c}}_{\text{i}}$ and ${\text{g}}_{\text{j}}$, respectively. Then the ARI can be calculated as follows:

(16) $$\text{ARI}=\frac{\sum_{\text{i},\text{j}}\left(\begin{array}{c} n_{ij}\\ 2 \end{array}\right)-\frac{\left[\sum_{i}\left(\begin{array}{c} n_{i.}\\ 2 \end{array}\right)\sum_{j}\left(\begin{array}{c} n_{.j}\\ 2 \end{array}\right)\right]}{\left(\begin{array}{c} n\\ 2 \end{array}\right)}}{\frac{1}{2}\left[\sum_{i}\left(\begin{array}{c} n_{i.}\\ 2 \end{array}\right)+\sum_{j}\left(\begin{array}{c} n_{.j}\\ 2 \end{array}\right)\right]-\frac{\left[\sum_{i}\left(\begin{array}{c} n_{i.}\\ 2 \end{array}\right)\sum_{j}\left(\begin{array}{c} n_{.j}\\ 2 \end{array}\right)\right]}{\left(\begin{array}{c} n\\ 2 \end{array}\right)}}.$$

Note that ARI will take a value between -1 and 1 and a higher value represents that two clusters are of more similarity. If the ARI value is 1, it means that the two clusters in comparison are the same.

## Competing interests

The authors declare that they have no competing interests.

## Authors' contributions

JX, XS and JG designed the framework of the proposed method. XS and JG constructed and implemented the method, and performed simulation experiments. JX designed the breast cancer study, and XS performed the data analysis with the help from JG and XC. AS, LHC and RC provided their biological interpretations on the breast cancer results. XS, JG and JX wrote and revised the manuscript. All authors read and approved the final manuscript.

## Declarations

The publication costs for this article were funded by National Institute of Health (NIH) [CA149653].

This article has been published as part of *BMC Systems Biology *Volume 7 Supplement 5, 2013: Selected articles from the International Conference on Intelligent Biology and Medicine (ICIBM 2013): Systems Biology. The full contents of the supplement are available online at http://www.biomedcentral.com/bmcsystbiol/supplements/7/S5.
